# White matter pathology – an endophenotype for bipolar disorder?

**DOI:** 10.1186/1471-244X-12-138

**Published:** 2012-09-13

**Authors:** Stefan Borgwardt, Paolo Fusar-Poli

**Affiliations:** 1 Department of Psychiatry, University of Basel, Basel, Switzerland; 2 King’s College, Institute of Psychiatry, King’s College London, London, UK; 3 Medical Image Analysis Centre, UniversityÂ of Basel, Basel, Switzerland

## Abstract

**Background:**

Neuroimaging investigations of white matter abnormalities in subjects at genetic risk for bipolar disorders (BD) potentially predating the onset of BD offer several advantages. They are not confounded by the presence of illness duration or previous treatment with medication and may ultimately inform evaluation of risk for subsequent development of BD and subsequent therapeutic intervention.

**Discussion:**

Although a number of imaging studies in subjects at genetic risk for BD are available the results are conflicting and no reliable structural markers of genetic liability to bipolar disorders have been proposed. We debate that white matter pathology may be central to the genetic risk to develop BD. Thus, white matter abnormalities detectable in HR subjects but not in controls may reflect genetically driven trait markers. Similar abnormalities may be also evident both in the HR and in BD, suggesting the possibility of genetic risk factors shared by both groups. Conversely, white matter alterations observed in BD patients but not in HR and controls can be interpreted as state markers.

**Summary:**

We suggest that white matter alterations may represent endophenotypes and neurobiological markers intermediate between the underlying susceptibility genes and the clinical expression of BD.

## Background

A number of structural brain imaging studies and meta-analytic reviews have shown that white matter reductions are consistently found in bipolar disorder [1]. A meta-analysis of 98 structural imaging studies showed that bipolar disorder was associated with lateral ventricle enlargement and increased rates of deep white matter hyperintensities [2]. Moreover, in a recently published tract-specific diffusion-tensor imaging (DTI) study by Benedetti et al. it was shown that patients with BD had significantly decreased average fractional anisotropy (FA) and increased average mean diffusivity in the majority of the white matter (WM) fiber bundles [3].

## Discussion

Neuroimaging investigations of structural and functional brain abnormalities in subjects at genetic risk for bipolar disorder (BD) offer several advantages. They are not confounded by illness duration or exposure to previous treatments and may ultimately inform evaluation of risk for subsequent development of BD and subsequent therapeutic intervention [4]. Although a number of imaging studies in subjects at high risk (HR) for BD are available, the results are conflicting and no reliable imaging markers of genetic liability to BD is clinically available yet [5-8].

In a recent systematic meta-analysis of neuroimaging studies of 996 HR subjects for BD and 1258 controls, we confirmed inconclusive structural findings but increased neurofunctional response during cognitive tasks in the prefrontal and temporo-insular regions [9]. On the basis of the study by Benedetti et al. [3] it is possible to speculate that temporo-limbic white matter (WM) alterations may alter the neurofunctional connectivity and ultimately the regional response during cognitive tasks. Specifically, region-of-interest magnetic resonance imaging (MRI) studies of twins found decreased left hemispheric WM volume in either BD patients or co-twins (HR) compared with control twin subjects [10]. This indicated that genes involved in the etiology of BD may contribute to the WM decreases found in BD patients and in their co-twins (HR) (trait markers) [11]. However, other MRI studies reported contrasting findings with no significant WM alterations in the HR as compared to controls [12,13] or to BD [14]. Others suggested more circumscribed and limited WM alterations in the major tracts of the brain [15] in line with the findings of Benedetti et al. [3]. DTI studies in the HR group showed reduced FA in the superior longitudinal fasciculus as compared to controls [6] and FA in HR intermediate to controls and BD patients [16]. Significant reductions in the number, density, and size of glial cells could be reflected in reduced WM tissue and signal hyperintensities, which are often reported in HR subjects [6].

The genetics of bipolar disorder is complex and relatives are most likely to carry some risk allels [11], unaffected relatives of BD patients are likely to share some susceptibility genes with affected patients. We therefore suggest that WM alterations may represent endophenotypes and neurobiological markers intermediate between the underlying susceptibility genes and the clinical expression of BD. A recently published study of monozygotic and dizygotic twin pairs revealed segregating genetic liabilities specifically associated with thicker right parietal cortex in schizophrenia and larger intracranial volume for BD [7]. However, disruptions in WM integrity have been implicated as endophenotype in schizophrenia and evidence includes neuroimaging studies of first-episode and chronic patients that report WM volume reductions and structural abnormalities, as well as myelin-related gene abnormalities [17]. Overlapping WM alterations between these two HR groups raises the possibility that schizophrenia and bipolar disorders share common endophenotypes [18-21].

## Summary

We suggested that white matter alterations may represent endophenotypes and neurobiological markers intermediate between the underlying susceptibility genes and the clinical expression of BD. Disturbed WM integrity in patients with BD will be better elucidated by imaging studies, investigating larger and more homogenous samples and employing longitudinal designs to dissect neurobiological abnormalities that are underlying traits of the illness from those related to psychopathological states such as episodes of mood exacerbation or pharmacological treatment. Moreover, there should be more direct comparisons between HR schizophrenia and HR BD groups. Similar to clinical [22,23], cognitive [24] and imaging [25-28] HR studies for schizophrenia, biomarkers of risk that differentiate those who will go on to develop BD from those that will not are needed. Additionally, future tractography studies following up genetic versus clinical HR samples are required to elucidate these issues and eventually clarify the role played by WM alterations in the development of the diseases.

## Competing interests

The authors declare that they have no competing interest.

## Authors’ contribution

SB and PFP drafted the manuscript. Both authors approved the final version.

## Authors’ information

Paolo Fusar-Poli is an Associate Professor at the Institute of Psychiatry, King's College, London, UK. Stefan Borgwardt is Professor of Neuropsychiatry at the University of Basel, Switzerland and Visiting Professor at the Institute of Psychiatry, King's College, London, UK.

## Pre-publication history

The pre-publication history for this paper can be accessed here:

http://www.biomedcentral.com/1471-244X/12/138/prepub
